# Multidrug‐Resistant *Providencia rettgeri*: An Emerging Pathogen in Farmed Siamese Crocodiles (*Crocodylus siamensis*) in Hainan, China

**DOI:** 10.1155/tbed/5761973

**Published:** 2026-08-26

**Authors:** Mengqi Wang, Caike Li, Yixin Tian, Zhongtao Liu, Shuai Ye, Jiahao Yu, Guiying Guo, Lixia Fan, Nuo Yang, Jifeng Zeng, Jiping Zheng, Xuesong Li

**Affiliations:** ^1^ Lab of Microbiological Engineering (Infection and Immunity), School of Life and Health Sciences, Hainan Province Key Laboratory of One Health, Collaborative Innovation Center of Life and Health, Hainan University, Haikou, Hainan, 570228, China, hainu.edu.cn; ^2^ School of Life and Health Sciences, Institute of Biomedical Research, Collaborative Innovation Center of Life and Health, Hainan University, Haikou, Hainan, 570228, China, hainu.edu.cn; ^3^ Dongfang Hewangxin Breeding Co. Ltd., Dongfang 572623, China; ^4^ School of Chemistry and Chemical Engineering, Hainan University, Haikou, Hainan, 570228, China, hainu.edu.cn; ^5^ Hainan International One Health Institute, Hainan University, Haikou, Hainan, 570228, China, hainu.edu.cn

**Keywords:** *Crocodylus siamensis*, disease surveillance, multidrug resistance, *Providencia*, virulence, zebrafish challenge

## Abstract

The Siamese crocodile (*Crocodylus siamensis*) farming industry in China, particularly in Hainan Province, has grown substantially, yet it faces increasing threats from infectious diseases. *Providencia* spp., especially *P. rettgeri*, are increasingly reported in reptilian disease outbreaks, but their epidemiological and pathogenic profiles in farmed *C. siamensis* remain poorly understood. In this study, necropsy and histopathological examination of 18 deceased juvenile Siamese crocodiles from three farms in Hainan Province, China, revealed severe multiorgan lesions indicative of septicemia. Sixty‐five *Providencia* isolates were recovered and assigned to four species‐level clusters (*P. rettgeri*, *P. stuartii*, *P. hangzhouensis*, and *P. alcalifaciens*) via 16S rDNA and *fusA* phylogenetic analyses. Most isolates exhibited strong motility but weak biofilm‐forming ability. Crucially, all 65 isolates were multidrug‐resistant and carried diverse antimicrobial resistance genes (ARGs), with 74% harboring class 1 integrons. Virulence‐associated genes (VGs) were widespread. Zebrafish challenge assays confirmed that all five representative isolates were pathogenic, with *P. rettgeri* strains PL‐425 and PL‐688 exhibiting the highest virulence (100% mortality within 24–30 h). Whole‐genome sequencing of PL‐425 identified multiple virulence loci related to motility, capsule, adhesion, and toxins. These findings identify multidrug‐resistant *P. rettgeri* as a dominant and highly virulent pathogen in farmed Siamese crocodiles, highlighting its emergence as a significant threat to the industry and underscoring the urgent need for integrated surveillance and antimicrobial stewardship.

**Summary**

• Sixty‐five *Providencia* isolates recovered from multiple organs of deceased juvenile Siamese crocodiles (*Crocodylus siamensis*) were classified into four species‐level clusters based on combined 16S rDNA and *fusA* phylogenetic analyses.
• All crocodile‐derived *Providencia* isolates were multidrug‐resistant, carried diverse antimicrobial resistance genes (ARGs), and showed a high prevalence of class 1 integrons.
• Zebrafish challenge and genomics revealed a highly virulent *P. rettgeri* strain with multiple virulence loci, highlighting its pathogenic threat to farmed Siamese crocodiles.

## 1. Introduction

Crocodile farming is an established aquaculture sector with considerable economic importance, while intensive farming conditions may increase the risk of infectious disease outbreaks [1, 2]. Hainan Province is an important region for captive Siamese crocodile farming in southern China. However, the intensification of farming systems has increased the risk of infectious diseases, posing direct threats to animal welfare, productivity, and the sustainable development of the industry. *Providencia rettgeri* has been reported in severe disease outbreaks in crocodilians, including meningitis in hatchling saltwater crocodiles and septicemia in farmed American alligators [3–5]. More recently, an outbreak of *P. rettgeri* bacteremia was reported in farmed rat snakes, further supporting the relevance of this bacterium as an emerging pathogen in farmed reptiles [6]. Therefore, the pathogenicity, antimicrobial resistance, and epidemiological characteristics of *P. rettgeri* and other *Providencia* species in farmed reptiles warrant further investigation.

Members of the genus *Providencia* are Gram‐negative, facultatively anaerobic opportunistic pathogens in the family Morganellaceae (formerly classified within Enterobacteriaceae) and have been associated with urinary tract infections, wound infections, bacteremia, and gastroenteritis in humans and animals [7, 8]. Among them, *Providencia alcalifaciens* has been most consistently linked to diarrheal disease and has been shown to possess invasion‐associated enteropathogenic potential [9–11]. In addition, the taxonomy of the genus *Providencia* has expanded substantially in recent years and remains under active revision, highlighting the importance of accurate species‐level identification when comparing isolates from different hosts or geographic regions [7, 12].

Although *Providencia*‐associated disease has been reported in crocodilians, including mixed infection in Chinese alligators, information on the phenotypic diversity, virulence heterogeneity, antimicrobial resistance, and genomic characteristics of *Providencia* isolates from farmed Siamese crocodiles (*Crocodylus siamensis*) in tropical regions remains limited [5, 13]. In the present study, we conducted an epidemiological investigation of deceased juvenile Siamese crocodiles from three major farming regions in Hainan Province, China, and compared the biological characteristics, antimicrobial resistance profiles, and virulence‐associated genotypes of the recovered *Providencia* strains. A hypervirulent isolate was further selected by zebrafish challenge and subjected to whole‐genome analysis to explore the genetic basis underlying its pathogenicity. This study provides new evidence for understanding the diversity and pathogenic potential of *Providencia* in crocodile farming and offers a scientific basis for disease surveillance, diagnosis, and control in this industry.

## 2. Materials and Methods

### 2.1. Collection, Autopsy, and Bacterial Isolation and Identification of Deceased Crocodiles

#### 2.1.1. Sample Collection and Dissection

Eighteen deceased juvenile Siamese crocodiles (*C. siamensis*) were collected from four farms in Dongfang City, one farm in Chengmai County, and one farm in Baisha Li Autonomous County, Hainan Province, China, between October 2022 and March 2024. All sample collections were conducted with the verbal informed consent of the farm owners, who approved the use of deceased crocodile tissues for noncommercial research purposes. The crocodiles were transported to the Laboratory of Infection and Immunology at Hainan University within 24 h postmortem for necropsy and pathological examination. Dissection was performed following the method described by Rehman et al. [14]. Briefly, the surface of the crocodile was thoroughly disinfected with iodine tincture in a biological safety cabinet. Before external gross lesions were recorded and photographed, residual iodine tincture was thoroughly removed from the body surface using sterile gauze to avoid potential discoloration artifacts. A midline incision was then made along the abdomen to examine gross pathological changes, and sterile samples of major organs (brain, heart, liver, kidney, spleen, and lung) were collected.

#### 2.1.2. Histopathology and Bacterial Isolation and Culture

Histopathological examination and bacterial isolation were performed as described previously for bacterial disease investigations in Siamese crocodiles, with minor modifications [14]. Each collected major organ from the deceased crocodiles was divided into two parts: one part was fixed in 4% paraformaldehyde (PFA) solution for 4 h and then sent to Wuhan Saville Biotechnology Co., Ltd. for paraffin embedding, sectioning, and staining. Stained slides were observed under a light microscope, with histopathological examinations performed at 10× and 100× magnifications.

For each of the 18 deceased crocodiles, bacterial isolation was attempted from a standardized panel of major organs and tissues, including heart blood, liver, spleen, kidney, lung, brain, eye, intestine, and stomach. Lesion‐associated fluids, including thoracic effusion and pericardial effusion, were also sampled when present. A tissue was recorded as culture‐negative when no *Providencia* isolate was recovered from that tissue under the culture conditions used in this study. A sterile inoculating loop was used to collect material from the interior of each organ and tissue, which was then streaked onto brain heart infusion (BHI) agar plates for bacterial isolation. The plates were incubated at 37°C for 24 h in a constant‐temperature incubator. Subsequently, single colonies with different morphologies were picked from the plates and subcultured on the same medium for purification. Finally, the purified bacteria were cultured in BHI broth for further studies.

#### 2.1.3. Bacterial Identification

Genomic DNA of the above strains was extracted using a genomic DNA extraction kit (Nanjing Novozan Biotechnology Co., Ltd., China). The 16S rDNA and *fusA* genes of each isolate were amplified by PCR (primer sequences are listed in Supporting Information 1: Table S1), and the PCR amplicons were sent to Guangzhou Tianyi Huiyuan Biotechnology Co., Ltd., China, for gene sequencing. The 16S rDNA sequences were compared with reference sequences in the database using the NCBI‐BLAST online tool to preliminarily determine the classification of each strain. Phylogenetic analysis based on 16S rDNA and *fusA* sequences was performed using MEGA X software (reference sequences of standard strains were obtained from the NCBI database). A phylogenetic tree was constructed using the neighbor‐joining method with a bootstrap value of 1000. The iTOL v.4.5 program was used for visualization and integration of available metadata into the phylogenetic tree.

### 2.2. Phenotypic Characterization of Bacterial Isolates

#### 2.2.1. Biofilm Formation Assay

Biofilm formation was determined by the crystal violet staining method according to Peeters et al. [15], with minor modifications. Activated bacterial cultures were adjusted to OD_600_ = 1.0 and diluted 1:100 in sterile LB broth. Then, 200 μL of each diluted culture was added to sterile 96‐well plates in triplicate, with sterile LB broth used as the negative control. After incubation at 37°C for 24 h, the cultures were discarded, and the wells were gently washed three times with sterile PBS and air‐dried. The attached cells were fixed with 200 μL methanol for 15 min, stained with 0.1% crystal violet for 15 min, washed three times, and air‐dried. The bound dye was then dissolved in 200 μL absolute ethanol, and the absorbance at 590 nm was measured after 15 min. The cut‐off value (ODc) was defined as the mean OD_590_ of the negative control plus three standard deviations. Biofilm formation was classified as follows: OD ≤ ODc, nonbiofilm producer; ODc < OD ≤ 2 × ODc, weak biofilm producer; 2 × ODc < OD ≤ 4 × ODc, moderate biofilm producer; and OD > 4 × ODc, strong biofilm producer.

#### 2.2.2. Motility Assay

Motility was assessed on semisolid LB agar plates according to the method of Chen et al. [16]. Activated bacterial cultures were adjusted to OD_600_ = 1.0. For swarming motility, 2 μL of each culture was spot‐inoculated onto the surface of LB medium containing 0.5% agar. For swimming motility, 2 μL of each culture was stab‐inoculated into the center of LB medium containing 0.3% agar. Four replicate plates were prepared for each isolate. The plates were incubated upright at 37°C for 24 h, after which the diameter of bacterial spread was recorded. Motility was classified based on the diffusion diameter after 24 h as follows: weak motility (*S*), <10 mm; moderate motility (*M*), 10–50 mm; and strong motility (*F*), >50 mm.

### 2.3. Antimicrobial Resistance Analysis

#### 2.3.1. Detection of Antimicrobial Resistance Genes (ARGs) and Class 1 Integrons

Primers targeting 10 common ARGs and the class 1 integron were designed based on nucleotide sequences retrieved from the NCBI database (Supporting Information 1: Table S1). Conventional PCR was performed to detect these targets in each isolate. The final reaction volume was 10 μL, containing 5 μL of 2× Taq Master Mix (Nanjing Novozan Biotechnology Co., Ltd., China), 0.5 μL each of forward and reverse primers, 1 μL of template DNA, and 3 μL of ddH_2_O. The amplification conditions were as follows: initial denaturation at 95°C for 3 min; 30 cycles of denaturation at 95°C for 15 s, primer‐specific annealing at the temperature listed in Supporting Information 1: Table S1 for 15 s, and extension at 72°C for 30 s; followed by a final extension at 72°C for 3 min. The PCR products were analyzed by 1% agarose gel electrophoresis at 100 V for 20 min and visualized using a gel documentation system.

#### 2.3.2. Antimicrobial Susceptibility Testing

##### 2.3.2.1. Disk Diffusion Assay

Antimicrobial susceptibility was evaluated using the disk diffusion method in accordance with the CLSI M02 standard, and inhibition zone diameters were interpreted using the applicable criteria provided in CLSI M100. Activated bacterial cultures were adjusted to a 0.5 McFarland standard (~1 × 10^8^ CFU/mL), and 100 μL of each suspension was evenly spread onto Mueller–Hinton (MH) agar plates. After the inoculum had dried, disks containing 20 antimicrobial agents representing β‐lactams, aminoglycosides, quinolones, tetracyclines, and other classes were placed on the agar surface. Four replicate plates were prepared for each isolate. After incubation at 37°C for 18 h, the diameters of the inhibition zones were measured, and the susceptibility results were interpreted according to CLSI guidelines.

##### 2.3.2.2. Minimum Inhibitory Concentration (MIC) Determination

The MICs of polymyxin B and tigecycline were determined by the broth microdilution method according to CLSI M07 and ISO 20776‐1 [17, 18, 19]. MIC results were interpreted with reference to CLSI M100, where applicable [20]. Freshly prepared MH broth was used for tigecycline testing to minimize the effect of oxidative degradation on MIC determination [21]. Activated bacterial cultures were adjusted to a 0.5 McFarland standard and then diluted 1:100 in fresh MH broth. Stock solutions of polymyxin B and tigecycline (5120 μg/mL) were diluted 1:10 to obtain working solutions of 512 μg/mL. Twofold serial dilutions were prepared in sterile 96‐well microtiter plates to yield final concentrations ranging from 0.5 to 256 μg/mL. Equal volumes of the bacterial suspension were added to each well. Wells containing inoculated broth without antibiotics served as the growth control, whereas wells containing sterile broth alone served as the blank control. The plates were incubated at 37°C for 16 h, and the OD_600_ values were measured using a microplate reader. The MIC was defined as the lowest antibiotic concentration showing no visible growth and an absorbance value comparable to that of the blank control. Each assay was performed in triplicate.

Multidrug resistance (MDR) was defined according to Magiorakos et al. [22] as acquired nonsusceptibility to at least one agent in three or more antimicrobial categories. Intermediate and resistant phenotypes were considered nonsusceptible for MDR classification. The MDR classification was based on the overall antimicrobial susceptibility profile across the tested antimicrobial categories and was not determined solely by intrinsic polymyxin B resistance in *Providencia* spp.

### 2.4. Pathogenicity Analysis

#### 2.4.1. Detection of Virulence Genes

Based on sequences retrieved from the NCBI database, specific primers targeting virulence‐associated genes (VGs) of *Providencia* spp. were designed (Supporting Information 1: Table S1). The target genes included fimbrial adhesion‐ and motility‐related genes (*fimC*, *papD*, *papC*, and *motA*), iron acquisition/metabolism‐related genes (*fepC*, *ireA*, and *fptA*), and outer membrane structure and efflux pump‐related genes (*ompA* and *acrA*). Conventional PCR was performed using the same reaction mixture and amplification conditions described above to determine the distribution of these virulence genes among the isolates.

#### 2.4.2. Zebrafish Challenge Test and Histopathological Analysis

##### 2.4.2.1. Zebrafish Husbandry and Animal Ethics

Healthy adult zebrafish were obtained from a local farm in Haikou, China, which holds valid ethical approval for the breeding and supply of laboratory animals, with a body weight of 0.35 ± 0.05 g. Fish were maintained in dechlorinated and oxygenated water at 28°C for at least 1 week before the experiment to allow acclimation to laboratory conditions, following the method described by Pu et al. [23]. All fish were fasted for 24 h prior to the experiment.

All animal procedures were conducted in accordance with the guidelines for the care and use of laboratory animals and were approved by the Animal Welfare and Ethics Committee of Hainan University (Approval Number HNUAUCC‐2023‐00166).

##### 2.4.2.2. Zebrafish Challenge Test and Histopathological Analysis

Five isolates showing strong biofilm‐forming ability, high motility, or carrying multiple virulence genes were selected from all isolates for the zebrafish challenge assay, including two *P. rettgeri* strains and one strain each of *Providencia stuartii*, *P. alcalifaciens*, and *Providencia hangzhouensis*. Activated bacterial cultures were adjusted to 1 × 10^7^ CFU/mL with sterile PBS. Zebrafish were briefly anesthetized by immersion in ice‐cold water (0–4°C) for ~3 s, and anesthesia was confirmed by a transient loss of the righting reflex and a markedly reduced response to gentle touch. Zebrafish were briefly anesthetized in ice water for ~3 s and then intraperitoneally injected with 10 μL of the bacterial suspension, corresponding to an infection dose of ~1 × 10^5^ CFU per fish. Fish in the control group were injected intraperitoneally with 10 μL sterile PBS. Each group contained 30 fish. Clinical signs and mortality were recorded every 6 h for 72 h postinfection. To confirm that zebrafish mortality was associated with the inoculated bacterial strains, freshly dead zebrafish from each infected group were aseptically dissected. Internal tissues were homogenized in sterile PBS, streaked onto BHI agar plates, and incubated at 37°C for 24 h. Representative colonies were purified and identified by 16S rDNA and/or *fusA* sequencing. The recovered isolates were confirmed to be consistent with the corresponding inoculated *Providencia* strains. No *Providencia* isolate was recovered from the PBS‐injected control group.

Dead zebrafish from each infected group and euthanized zebrafish from the control group (*n* = 5) were homogenized for bacterial isolation and identification to confirm *Providencia* infection. To comply with journal requirements for vertebrate animal studies, zebrafish designated for sampling (including control fish) were humanely euthanized using an approved method consistent with institutional animal care guidelines and applicable veterinary recommendations. Specifically, fish were euthanized by overdose immersion in buffered tricaine methanesulfonate (MS‐222; 300 mg/L, sodium bicarbonate–buffered to pH 7.0–7.5) until cessation of opercular movement for ≥10 min, followed by confirmation of death by the absence of heartbeat and a secondary physical method (e.g., decapitation or severing the spinal cord). In addition, three dead zebrafish from the *P. rettgeri* PL‐425–challenged group and three euthanized zebrafish from the PBS‐injected control group were submitted to Wuhan Saville Biotechnology Co., Ltd. (Wuhan, China) for histopathological analysis.

### 2.5. Whole‐Genome Analysis of the Highly Virulent *P. rettgeri* Strain PL‐425

Genomic DNA of strain PL‐425 was extracted using a bacterial DNA extraction kit (Vazyme, China) according to the manufacturer’s instructions. Whole‐genome sequencing was performed by Meiji Biotechnology Co., Ltd. (Guangzhou, China). Raw reads were quality‐filtered by trimming bases with quality scores ≤ Q20 at both ends, removing reads containing adapter sequences (adapter match length ≥ 15 bp), and discarding reads containing ≥ 5 ambiguous bases (*N*). Read quality, GC content, and sequence duplication levels were assessed using FastQC v0.11.9 [24], and the resulting clean reads were used for downstream analyses. De novo genome assembly was performed using SPAdes [25], followed by gap filling with GapCloser [26]. Assembly quality and completeness were evaluated using QUAST [27] and BUSCO [28], respectively.

For genome annotation and feature analysis, coding sequences were predicted using Glimmer3 [29]. Noncoding RNAs (ncRNAs) were identified using tRNAscan‐SE [30], RNAmmer [31], and the Rfam database [32]. Interspersed repeat sequences were identified using RepeatMasker [33], while prophage regions and genomic islands (GIs) were predicted using Phage_Finder [34] and IslandPath‐DIMOB [35], respectively. CRISPR arrays were identified using CRISPRdigger [36]. Functional annotation was performed against the NCBI nonredundant protein (NR) [37] and Swiss‐Prot [38] databases. COG functional classification was conducted based on the eggNOG database [39], and Gene Ontology (GO) analysis was used for functional categorization [40]. Metabolic pathways were annotated using the Kyoto Encyclopedia of Genes and Genomes (KEGG) database [41], and carbohydrate‐active enzymes were predicted using the CAZy database [42]. Finally, a circular genome map was generated using Circos [43] to visualize genomic features, including gene distribution, COG functional categories, GC content, and GIs.

## 3. Results

### 3.1. Gross and Histopathological Findings in Dead Juvenile Crocodiles

#### 3.1.1. Gross Pathological Findings

**Figure 1:**
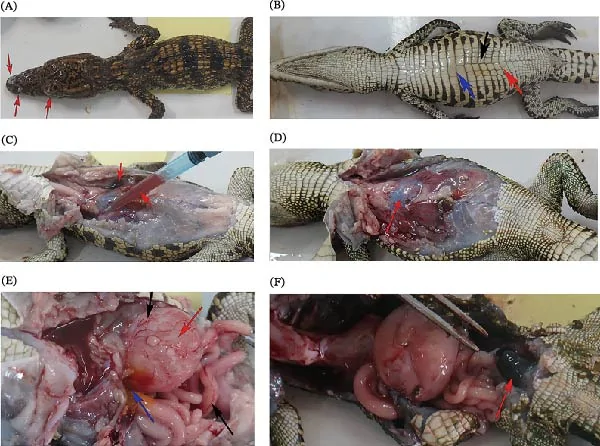
Gross clinical lesions in different organs of dead juvenile Siamese crocodiles. (A) Milky white mucus was observed around the nasal cavity and eyelids (red arrows). (B) Gross external lesions included gray‐brown ecchymotic discoloration on the ventral scales (blue arrow), which was photographed after residual iodine tincture had been thoroughly removed from the body surface; localized yellow‐brown discoloration of the scales over the gallbladder region was interpreted cautiously as possible bile staining/imbibition rather than as an independent antemortem lesion (black arrow); marked abdominal distension was also observed (red arrow). (C) Visible fluid accumulation was present in the thoracic cavity, and the fluid volume was estimated to exceed 1 mL based on gross necropsy observation (red arrows). (D) The pericardium was markedly distended and contained abundant fluid (red arrow). (E) Gross lesions of the digestive organs included severe gastric distension (red arrow); after incision, the stomach contained gas and viscous fluid, indicating intraluminal gas and fluid accumulation. Congestion/hyperemia was observed on the serosal surfaces of the stomach and intestine (black arrow). The gallbladder appeared intact and filled with bile, and orange‐brown staining on the adjacent gastric serosa was interpreted cautiously as possible postmortem bile imbibition rather than gallbladder rupture (blue arrow). (F) External darkening of the rectum was observed (red arrow). Rectal obstruction was confirmed after incision of the affected segment by the presence of black, hardened solid intraluminal material within the rectal lumen.

The dead juvenile crocodiles weighed 52.13–269.53 g and measured 18.2–42.3 cm in snout‐to‐vent length. Some dead crocodiles exhibited large amounts of milky white mucus in the nasal cavity and on the eyelids (Figure 1A, red arrows). Gray‐brown ecchymotic discoloration was observed on the ventral scales of the examined dead crocodiles (Figure 1B, blue arrows). These lesions were recorded after residual iodine tincture had been carefully removed from the body surface. Localized yellow‐brown discoloration was observed on the scales over the gallbladder region (Figure 1B, black arrows) and was interpreted cautiously as possible bile staining/imbibition rather than as an independent antemortem lesion. Marked abdominal distension was also observed (Figure 1B, red arrows).

Necropsy further revealed visible fluid accumulation in the thoracic cavity (thoracic/pleural effusion; Figure 1C, red arrows). Although the effusion was not measured volumetrically during the original necropsy, the visible fluid volume was estimated to exceed 1 mL in the affected crocodiles. The pericardium was markedly distended and contained abundant fluid (Figure 1D, red arrows). In all dead crocodiles, the stomach was severely distended (Figure 1E, red arrows). After incision of the distended stomach, the gastric lumen was found to contain gas and viscous fluid, indicating that the gastric distension was mainly associated with intraluminal gas and fluid accumulation. The serosal surfaces of the stomach and intestine appeared congested/hyperemic (Figure 1E, black arrows). The gallbladder appeared intact and filled with bile, and orange‐brown staining was observed on the adjacent gastric serosa (Figure 1E, blue arrows). This finding was interpreted cautiously as possible postmortem bile imbibition rather than gallbladder rupture or an independent antemortem lesion. Darkening of the rectum was observed externally (Figure 1F, red arrows). After incision of the affected rectal segment, black, hardened solid intraluminal material was found within the rectal lumen, confirming the presence of rectal obstruction.

#### 3.1.2. Histopathological Findings

**Figure 2:**
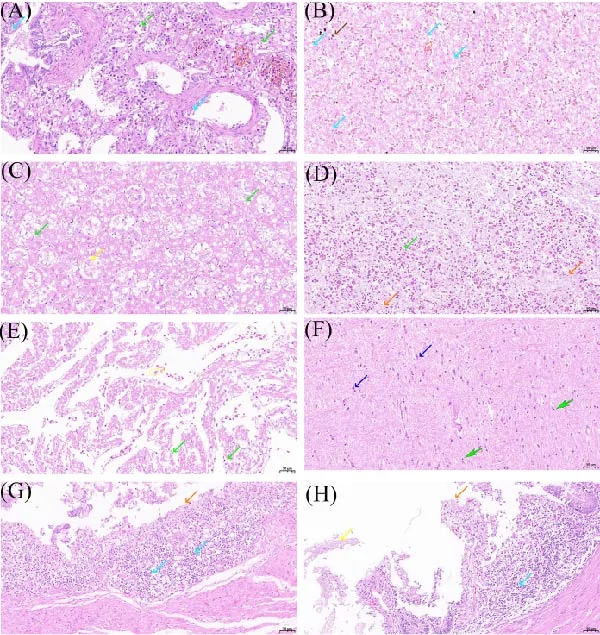
Histopathological lesions in different organs of dead juvenile Siamese crocodiles. Histopathological examination of major organs from dead juvenile Siamese crocodiles. Sections were stained with hematoxylin and eosin (H&E); scale bars = 50 μm. (A) Lung: moderate to severe widening/thickening of the alveolar/faveolar walls and septa, with abundant inflammatory cell infiltration within the alveolar/faveolar walls and lumina (green arrows). Mild edema and swelling of bronchial epithelial cells with pale, loose cytoplasm are indicated by cyan arrows. A small amount of eosinophilic material in the bronchial lumen is outlined by a silver polygon, and rare vascular congestion is outlined by an orange polygon. (B) Liver: abundant lipid droplets and mild hepatocellular swelling (cyan arrows), with scattered brown‐yellow pigment deposition (brown arrows). (C) Kidney: extensive sloughing of renal tubular epithelial cells (yellow arrows) accompanied by vacuolar degeneration (green arrows). (D) Spleen: indistinct red and white pulp architecture, reduced lymphocytes in the red pulp, abundant necrotic cellular debris (orange arrows), and mild inflammatory cell infiltration (green arrows). (E) Heart: loosely arranged and irregular myocardial fibers with mild hemorrhage (yellow curved polygon) and sparse inflammatory cell infiltration (green arrows). (F) Brain: occasional neuronal atrophy (blue arrows) and mild interstitial inflammatory cell infiltration (green arrows). (G) Cecum: extensive sloughing of the mucosal epithelium with exposure of the lamina propria (orange arrows), marked lymphocytic infiltration in the lamina propria, reduced intestinal glands, and relaxation of the smooth muscle layer. (H) Duodenum: focal villous necrosis with karyolysis (yellow arrows), extensive villous epithelial sloughing (orange arrows), exposure of the lamina propria, marked lymphocytic infiltration (cyan arrows), and reduced intestinal glands.

Pulmonary lesions were characterized by moderate to severe widening/thickening of the alveolar/faveolar walls and septa, with abundant inflammatory cell infiltration within the alveolar/faveolar walls and lumina (Figure 2A, green arrows). Mild edema and swelling of bronchial epithelial cells, characterized by pale and loose cytoplasm, were occasionally observed (Figure 2A, cyan arrows). A small amount of eosinophilic material was present within the bronchial lumen (Figure 2A, silver‐outlined polygon), and vascular congestion was rarely observed (Figure 2A, orange‐outlined polygon). Considering the normal prominence of connective tissue in reptilian pulmonary faveolar walls, the apparent septal thickening was interpreted as a pathological change mainly associated with inflammatory cell infiltration and septal widening, with occasional epithelial edema and limited vascular congestion, rather than as fibrosis or nonspecific thickening alone.

Hepatic changes included abundant lipid droplets within hepatocytes, mild hepatocellular swelling (Figure 2B, cyan arrows), and scattered brown‐yellow pigment deposition (Figure 2B, brown arrows). Renal lesions were characterized by extensive sloughing of renal tubular epithelial cells (Figure 2C, yellow arrows) with vacuolar degeneration (Figure 2C, green arrows).

Splenic lesions included an indistinct red and white pulp architecture, reduced lymphocytes in the red pulp, abundant necrotic cellular debris (Figure 2D, orange arrows), and mild inflammatory cell infiltration (Figure 2D, green arrows). In the heart, myocardial fibers were loosely arranged and irregularly oriented, with mild hemorrhage (Figure 2E, yellow curved polygon) and sparse inflammatory cell infiltration (Figure 2E, green arrows). Occasional neuronal atrophy was observed in the brain (Figure 2F, blue arrows), together with mild interstitial inflammatory cell infiltration (Figure 2F, green arrows). However, no definitive histopathological evidence of meningitis, such as meningeal thickening, meningeal inflammatory cell infiltration, or inflammatory exudate within the meningeal/submeningeal space, was observed in the examined brain sections.

Cecal lesions were characterized by extensive sloughing of the mucosal epithelium with exposure of the lamina propria (Figure 2G, orange arrows), marked lymphocytic infiltration in the lamina propria, reduced intestinal glands, and relaxation of the smooth muscle layer. Duodenal lesions included focal villous necrosis with karyolysis (Figure 2H, yellow arrows), extensive villous epithelial sloughing (Figure 2H, orange arrows), exposure of the lamina propria, marked lymphocytic infiltration (Figure 2H, cyan arrows), and reduced intestinal glands.

### 3.2. Isolation and Species Identification of *Providencia* Isolates

**Figure 3:**
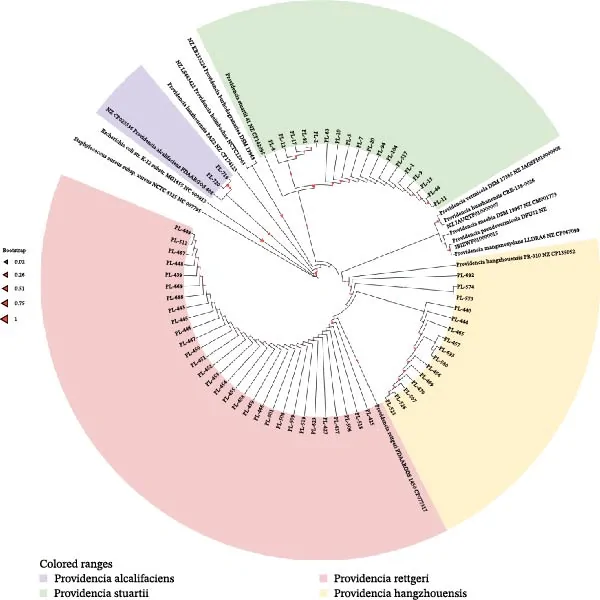
Circular phylogenetic tree of 65 *Providencia* isolates recovered from dead juvenile Siamese crocodiles based on combined 16S rDNA and *fusA* gene sequences. A circular phylogenetic tree was constructed based on combined 16S rDNA and *fusA* gene sequences of the 65 crocodile‐derived *Providencia* isolates together with reference strains retrieved from GenBank. The isolates were assigned to four species‐level clusters, corresponding to *Providencia alcalifaciens* (purple), *Providencia stuartii* (green), *Providencia rettgeri* (pink), and *Providencia hangzhouensis* (yellow). Bootstrap support values are indicated by red triangles at the nodes, with triangle size proportional to the bootstrap value. *Escherichia coli* K‐12 substr. MG1655 and *Staphylococcus aureus* subsp. *aureus* NCTC 8325 were included as outgroup taxa.

A total of 65 bacterial isolates were recovered from 18 dead juvenile crocodiles collected from different crocodile farms in Dongfang, Chengmai, and Baisha Li Autonomous County, Hainan Province, China. The isolates were obtained from multiple organs and tissues, including the liver, brain, heart blood, kidney, intestine, eye, lung, spleen, thoracic effusion, pericardial effusion, and stomach. *Providencia* was recovered from multiple organs or tissues in some crocodiles, whereas only one or a few culture‐positive tissues were detected in others. Therefore, the tissue distribution shown in Table 1 reflects differences in culture positivity among standardized samples rather than variation in the sampling protocol. Based on combined phylogenetic analysis of the 16S rDNA and *fusA* gene sequences, these isolates were assigned to four *Providencia* species–level clusters (Figure 3). Among them, *P. rettgeri* was the predominant species, accounting for 31 isolates (47.7%), followed by *P. stuartii* with 18 isolates (27.7%), *P. hangzhouensis* with 14 isolates (21.5%), and *P. alcalifaciens* with two isolates (3.1%) (Table 1).

**Table 1:** Isolation information of 65 *Providencia* strains recovered from 18 dead juvenile Siamese crocodiles in Hainan Province, China.

Isolate ID	Species	Sampling region	Crocodile ID	Isolation source
PL‐1	*Providencia stuartii*	Dongfang, Hainan, CHN	Crocodile1	Liver
PL‐2	*Providencia stuartii*	Dongfang, Hainan, CHN	Crocodile1	Liver
PL‐3	*Providencia stuartii*	Dongfang, Hainan, CHN	Crocodile1	Liver
PL‐7	*Providencia stuartii*	Dongfang, Hainan, CHN	Crocodile1	Brain
PL‐8	*Providencia stuartii*	Dongfang, Hainan, CHN	Crocodile1	Brain
PL‐9	*Providencia stuartii*	Dongfang, Hainan, CHN	Crocodile1	Brain
PL‐10	*Providencia stuartii*	Dongfang, Hainan, CHN	Crocodile1	Cardiac blood
PL‐11	*Providencia stuartii*	Dongfang, Hainan, CHN	Crocodile1	Cardiac blood
PL‐12	*Providencia stuartii*	Dongfang, Hainan, CHN	Crocodile1	Cardiac blood
PL‐13	*Providencia stuartii*	Dongfang, Hainan, CHN	Crocodile1	Kidney
PL‐17	*Providencia stuartii*	Dongfang, Hainan, CHN	Crocodile1	Intestinal tract
PL‐20	*Providencia stuartii*	Dongfang, Hainan, CHN	Crocodile1	Eye
PL‐44	*Providencia stuartii*	Dongfang, Hainan, CHN	Crocodile2	Liver
PL‐63	*Providencia stuartii*	Dongfang, Hainan, CHN	Crocodile3	Kidney
PL‐91	*Providencia stuartii*	Dongfang, Hainan, CHN	Crocodile4	Liver
PL‐94	*Providencia stuartii*	Dongfang, Hainan, CHN	Crocodile4	Kidney
PL‐104	*Providencia stuartii*	Dongfang, Hainan, CHN	Crocodile5	Intestinal tract
PL‐425	*Providencia rettgeri*	Dongfang, Hainan, CHN	Crocodile6	Eye
PL‐427	*Providencia rettgeri*	Dongfang, Hainan, CHN	Crocodile6	Brain
PL‐437	*Providencia rettgeri*	Dongfang, Hainan, CHN	Crocodile7	Kidney
PL‐439	*Providencia rettgeri*	Dongfang, Hainan, CHN	Crocodile7	Cardiac blood
PL‐440	*Providencia hangzhouensis*	Dongfang, Hainan, CHN	Crocodile7	Lung
PL‐443	*Providencia rettgeri*	Dongfang, Hainan, CHN	Crocodile7	Eye
PL‐444	*Providencia hangzhouensis*	Dongfang, Hainan, CHN	Crocodile7	Brain
PL‐445	*Providencia rettgeri*	Dongfang, Hainan, CHN	Crocodile8	Kidney
PL‐446	*Providencia rettgeri*	Dongfang, Hainan, CHN	Crocodile8	Kidney
PL‐447	*Providencia rettgeri*	Dongfang, Hainan, CHN	Crocodile8	Spleen
PL‐448	*Providencia rettgeri*	Dongfang, Hainan, CHN	Crocodile8	Spleen
PL‐449	*Providencia rettgeri*	Dongfang, Hainan, CHN	Crocodile8	Cardiac blood
PL‐450	*Providencia rettgeri*	Dongfang, Hainan, CHN	Crocodile8	Pleural effusion
PL‐451	*Providencia rettgeri*	Dongfang, Hainan, CHN	Crocodile8	Liver
PL‐452	*Providencia rettgeri*	Dongfang, Hainan, CHN	Crocodile8	Eye
PL‐453	*Providencia rettgeri*	Dongfang, Hainan, CHN	Crocodile8	Brain
PL‐454	*Providencia rettgeri*	Dongfang, Hainan, CHN	Crocodile9	Brain
PL‐455	*Providencia rettgeri*	Dongfang, Hainan, CHN	Crocodile9	Eye
PL‐456	*Providencia hangzhouensis*	Dongfang, Hainan, CHN	Crocodile9	Kidney
PL‐457	*Providencia hangzhouensis*	Dongfang, Hainan, CHN	Crocodile9	Cardiac blood
PL‐458	*Providencia rettgeri*	Dongfang, Hainan, CHN	Crocodile9	Spleen
PL‐459	*Providencia rettgeri*	Dongfang, Hainan, CHN	Crocodile9	Liver
PL‐465	*Providencia hangzhouensis*	Dongfang, Hainan, CHN	Crocodile10	Lung
PL‐466	*Providencia rettgeri*	Dongfang, Hainan, CHN	Crocodile10	Cardiac blood
PL‐467	*Providencia rettgeri*	Dongfang, Hainan, CHN	Crocodile10	Kidney
PL‐469	*Providencia rettgeri*	Dongfang, Hainan, CHN	Crocodile10	Eye
PL‐470	*Providencia hangzhouensis*	Dongfang, Hainan, CHN	Crocodile10	Liver
PL‐501	*Providencia rettgeri*	Dongfang, Hainan, CHN	Crocodile11	Liver
PL‐506	*Providencia rettgeri*	Dongfang, Hainan, CHN	Crocodile11	Kidney
PL‐507	*Providencia hangzhouensis*	Dongfang, Hainan, CHN	Crocodile11	Pericardial effusion
PL‐508	*Providencia rettgeri*	Dongfang, Hainan, CHN	Crocodile11	Spleen
PL‐509	*Providencia rettgeri*	Dongfang, Hainan, CHN	Crocodile11	Lung
PL‐511	*Providencia rettgeri*	Dongfang, Hainan, CHN	Crocodile11	Eye
PL‐517	*Providencia stuartii*	Dongfang, Hainan, CHN	Crocodile12	Kidney
PL‐518	*Providencia rettgeri*	Dongfang, Hainan, CHN	Crocodile12	Cardiac blood
PL‐519	*Providencia rettgeri*	Dongfang, Hainan, CHN	Crocodile12	Liver
PL‐523	*Providencia hangzhouensis*	Dongfang, Hainan, CHN	Crocodile12	Eye
PL‐526	*Providencia hangzhouensis*	Dongfang, Hainan, CHN	Crocodile12	Lung
PL‐573	*Providencia hangzhouensis*	Dongfang, Hainan, CHN	Crocodile13	Stomach
PL‐574	*Providencia hangzhouensis*	Dongfang, Hainan, CHN	Crocodile13	Kidney
PL‐580	*Providencia hangzhouensis*	Dongfang, Hainan, CHN	Crocodile13	Intestinal tract
PL‐623	*Providencia rettgeri*	Dongfang, Hainan, CHN	Crocodile14	Kidney
PL‐669	*Providencia rettgeri*	Dongfang, Hainan, CHN	Crocodile15	Liver
PL‐688	*Providencia rettgeri*	Dongfang, Hainan, CHN	Crocodile16	Brain
PL‐692	*Providencia hangzhouensis*	Baisha, Hainan, CHN	Crocodile17	Liver
PL‐693	*Providencia hangzhouensis*	Baisha, Hainan, CHN	Crocodile17	Liver
PL‐719	*Providencia alcalifaciens*	Chengmai, Hainan, CHN	Crocodile18	Intestinal tract
PL‐720	*Providencia alcalifaciens*	Chengmai, Hainan, CHN	Crocodile18	Intestinal tract

### 3.3. Biofilm Formation and Motility Characteristics

**Figure 4:**
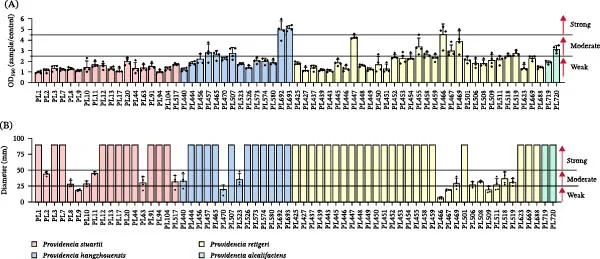
Distribution of biofilm‐forming ability and motility among 65 *Providencia* isolates recovered from dead juvenile Siamese crocodiles. (A) Biofilm‐forming ability of the isolates classified as weak, moderate, or strong based on the crystal violet staining assay. (B) Motility of the isolates classified as weak, moderate, or strong based on the semisolid agar assay.

Biofilm‐forming ability and motility were evaluated for all isolates using the crystal violet staining method and semisolid agar assay, respectively. The biofilm assay showed that most of the 65 *Providencia* isolates were weak biofilm producers (*n* = 39, 60.0%), whereas 22 isolates (33.8%) were moderate biofilm producers, and only four isolates (6.1%) were strong biofilm producers (Figure 4A). In contrast, motility testing showed that most isolates exhibited strong motility (*n* = 46, 70.8%), while 14 isolates (21.5%) showed moderate motility, and only five isolates (7.7%) showed weak motility (Figure 4B).

Further analysis by geographic origin and species revealed differences in biofilm formation and motility. Among the 61 isolates from Dongfang, three species were identified. All 18 *P. stuartii* isolates were weak biofilm producers; in terms of motility, one isolate (5.6%) showed weak motility, six isolates (33.3%) showed moderate motility, and 11 isolates (61.1%) showed strong motility. Among the 31 *P. rettgeri* isolates, 15 (48.4%) were weak biofilm producers, 14 (45.2%) were moderate biofilm producers, and two (6.5%) were strong biofilm producers; their motility profiles included three isolates (9.7%) with weak motility, six isolates (19.4%) with moderate motility, and 22 isolates (71.0%) with strong motility. Of the 12 *P. hangzhouensis* isolates from Dongfang, five (41.7%) were weak biofilm producers, and seven (58.3%) were moderate biofilm producers; their motility profiles included one isolate (8.3%) with weak motility, two isolates (16.7%) with moderate motility, and nine isolates (75.0%) with strong motility. The two *P. hangzhouensis* isolates from Baisha Li Autonomous County both exhibited strong biofilm‐forming ability and strong motility. The two *P. alcalifaciens* isolates from Chengmai showed moderate and weak biofilm‐forming ability, respectively, and both exhibited strong motility (Table 2).

**Table 2:** Distribution of biofilm‐forming ability and motility among *Providencia* strains isolated from different regions of Hainan, China.

City/state of isolation	Species	Number of isolates	Number of biofilm producers (%)			Number of strains classified by motility (%)		
			Weak	Moderate	Strong	Weak	Moderate	Strong
Dongfang, Hainan, CHN (*n* = 61)	*Providencia stuartii*	18	18 (100)	0	0	1 (5.6)	6 (33.3)	11 (61.1)
	*Providencia rettgeri*	31	15 (48.4)	14 (45.2)	2 (6.5)	3 (9.7)	6 (19.4)	22 (71.0)
	*Providencia hangzhouensis*	12	5 (41.7)	7 (58.3)	0	1 (8.3)	2 (16.7)	9 (75.0)
Baisha, Hainan, CHN (*n* = 2)	*Providencia hangzhouensis*	2	0	0	2 (100)	0	0	2 (100)
Chengmai, Hainan, CHN (*n* = 2)	*Providencia alcalifaciens*	2	1 (50.0)	1 (50.0)	0	0	0	2 (100)
Total		65	39 (60.0)	22 (33.8)	4 (6.1)	5 (7.7)	14 (21.5)	46 (70.8)

### 3.4. Antimicrobial Resistance Profiles

#### 3.4.1. Distribution of ARGs and Class 1 Integrons

**Figure 5:**
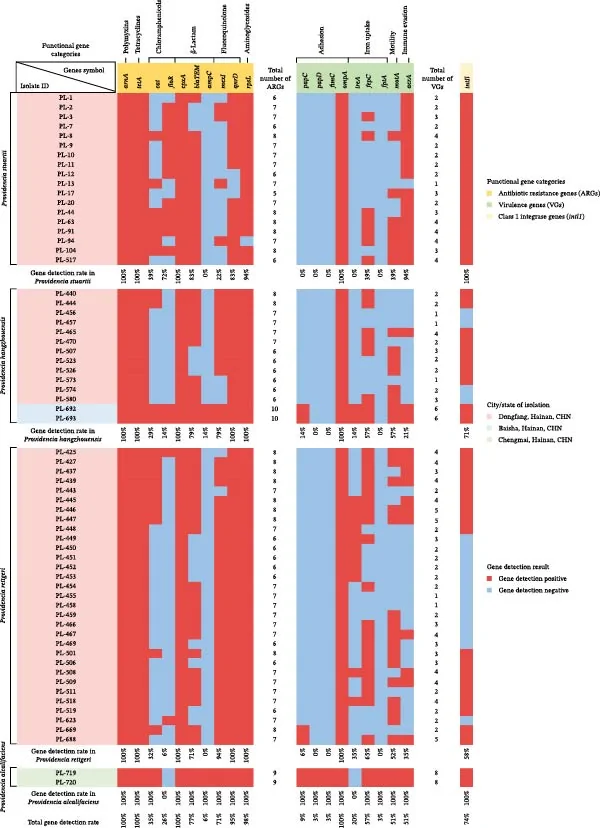
Distribution of antimicrobial resistance genes, virulence‐associated genes, and class 1 integrons among 65 *Providencia* isolates recovered from dead juvenile Siamese crocodiles. Heatmap showing the distribution of antimicrobial resistance genes (ARGs), virulence‐associated genes (VGs), and the class 1 integrase gene (*intI1*) among 65 *Providencia* isolates recovered from dead juvenile Siamese crocodiles. The isolates are grouped by species, including *Providencia stuartii*, *Providencia hangzhouensis*, *Providencia rettgeri*, and *Providencia alcalifaciens*. ARGs are classified into major functional categories, including polymyxins, tetracyclines, chloramphenicols, β‐lactams, fluoroquinolones, and aminoglycosides. VGs are categorized into adhesion, iron uptake, motility, and immune evasion–related genes. Red and blue boxes indicate positive and negative PCR detection, respectively. The columns “Total number of ARGs” and “Total number of VGs” indicate the number of detected genes in each isolate. The percentages shown below each species group and at the bottom indicate the detection rates of each gene within species and across all isolates, respectively.

A total of 65 *Providencia* isolates recovered from different organs of dead crocodiles from three regions of Hainan Province were screened for ARGs and class 1 integrons. Among all isolates, *arnA*, *tetA*, and *cpxA* were detected in all strains (100% and 65/65). In addition, *rpsL* and *qnrD* were highly prevalent, with detection rates of 98% (64/65) and 95% (62/65), respectively. *bla*_TEM_ and *mexI* were detected at intermediate frequencies, with positivity rates of 77% (50/65) and 71% (46/65), respectively, whereas *cat*, *floR*, and *ampC* were less common, with detection rates of 35% (23/65), 26% (17/65), and 6% (4/65), respectively. Class 1 integrons were detected in 74% (48/65) of the isolates. In terms of ARG burden, all isolates carried at least five of the 10 resistance targets examined, and some strains carried all 10 ARGs (Figure 5).

At the species and geographic levels, the distribution of ARGs varied among isolates. Among the 18 *P. stuartii* isolates from Dongfang, all targets except *mexI* were detected at frequencies above 80%, whereas *mexI* was detected in only 22% (4/18) of the isolates. These strains carried 5–8 ARGs, and all of them were positive for class 1 integrons (100%, 18/18). Among the 14 *P. hangzhouensis* isolates, the two strains from Baisha Li Autonomous County carried all 10 ARGs and were both positive for class 1 integrons (100%, 2/2), whereas the 12 strains from Dongfang carried 6–8 ARGs, with a class 1 integron positivity rate of 66% (8/12). Among the 31 *P. rettgeri* isolates from Dongfang, *mexI* was highly prevalent (94%, 29/31), whereas *ampC* was not detected in any isolate (0%, 0/31). These strains carried 6–8 ARGs, and 58% (18/31) were positive for class 1 integrons. The two *P. alcalifaciens* isolates from Chengmai carried nine ARGs each; all nine targets except *floR* were detected in both isolates (100%, 2/2), and both strains were positive for class 1 integrons (100%, 2/2) (Figure 5). Overall, ARG carriage was widespread among *Providencia* isolates from dead crocodiles in Hainan Province, with clear differences in ARG composition among species and geographic origins. The high prevalence of class 1 integrons further suggests their potential role in the dissemination of antimicrobial resistance determinants.

#### 3.4.2. Antimicrobial Susceptibility Testing Revealed Widespread MDR

Antimicrobial susceptibility testing was performed for the 65 *Providencia* isolates against 22 antibiotics representing multiple antimicrobial categories, including tetracyclines, phenicols, aminoglycosides, phosphonic acids, monobactams, quinolones/fluoroquinolones, cephalosporins, carbapenems, folate pathway inhibitors, β‐lactam/β‐lactamase inhibitor combinations, polymyxin B, and tigecycline. According to the criteria proposed by Magiorakos et al. [22], MDR was defined as nonsusceptibility to at least one antimicrobial agent in three or more antimicrobial categories. Based on this definition, all 65 isolates were classified as multidrug‐resistant.

Overall, the highest resistance rates were observed for polymyxin B (100%, 65/65), levofloxacin (91%, 59/65), and the three tetracyclines doxycycline, tetracycline, and oxytetracycline (each 91%, 59/65). Moderate‐to‐high resistance rates were also observed for chloramphenicol (51%, 33/65), streptomycin (51%, 33/65), ceftazidime (48%, 31/65), aztreonam (38%, 25/65), amikacin (34%, 22/65), and ampicillin‐sulbactam (31%, 20/65). In contrast, resistance to florfenicol was comparatively low (25%, 16/65), whereas resistance to imipenem (5%, 3/65), gentamicin (3%, 2/65), and nalidixic acid (2%, 1/65) was uncommon. When susceptibility was assessed, only five agents showed an overall susceptibility rate above 50%: gentamicin (92%, 60/65), tigecycline (88%, 57/65), imipenem (80%, 52/65), florfenicol (70%, 46/65), and ceftriaxone (64%, 42/65). Each isolate was resistant to at least four and up to 15 of the tested antibiotics, highlighting the severe antimicrobial resistance phenotypes of these strains. Notably, tigecycline susceptibility in *Providencia* should be interpreted with caution (Figure 6A).

Figure 6Antimicrobial susceptibility patterns of 65 *Providencia* isolates recovered from dead juvenile Siamese crocodiles. (A) Stacked bar chart showing the overall proportions of resistant, intermediate, and susceptible isolates for each tested antibiotic. (B) Heatmap of antimicrobial susceptibility profiles of individual isolates arranged by species. Red, yellow, and blue indicate resistant, intermediate, and susceptible phenotypes, respectively. The rightmost column indicates the total number of resistant antibiotics for each isolate, and the percentages shown below each species group represent species‐specific resistance rates for each antibiotic.

**Figure:**
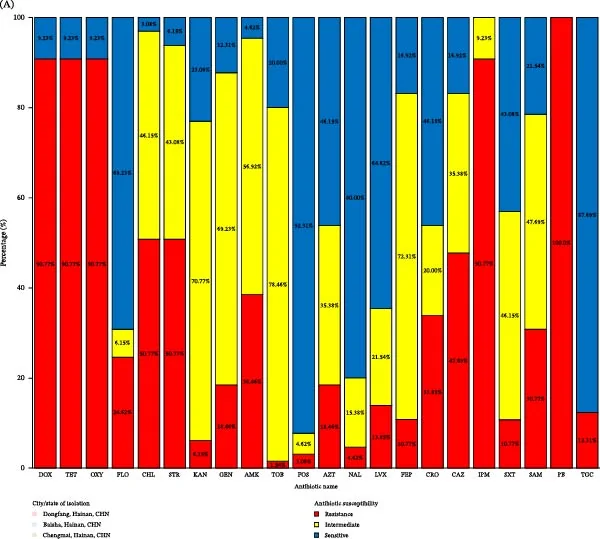

**Figure:**
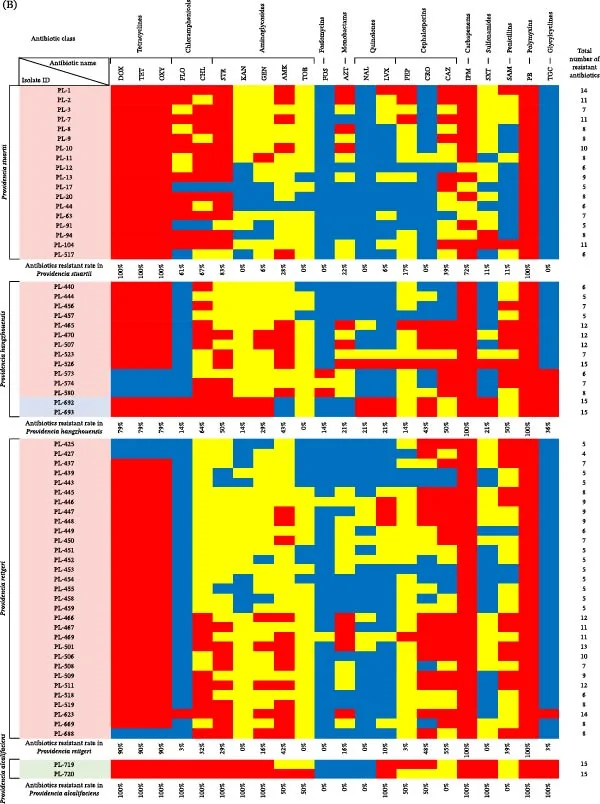

Marked species‐associated differences were observed in the resistance profiles. Among the 18 *P. stuartii* isolates from Dongfang, resistance to the three tetracyclines and polymyxin B was universal (100%, 18/18). High resistance rates were also observed for streptomycin (83%, 15/18), chloramphenicol (67%, 12/18), and florfenicol (61%, 11/18), whereas all isolates remained susceptible to gentamicin, tobramycin, cefepime, and imipenem. For the 14 *P. hangzhouensis* isolates (two from Baisha Li Autonomous County and 12 from Dongfang), resistance to polymyxin B and levofloxacin was universal (100%, 14/14), and tetracycline resistance remained high (79%, 11/14). The 31 *P. rettgeri* isolates from Dongfang showed high resistance to tetracyclines (90%, 28/31), levofloxacin (100%, 31/31), and polymyxin B (100%, 31/31) while remaining fully susceptible to imipenem and cefepime. The two *P. alcalifaciens* isolates from Chengmai exhibited the broadest resistance profiles, with 100% resistance to 15 antibiotics, including all three tetracyclines, both phenicols, streptomycin, kanamycin, fosfomycin, polymyxin B, levofloxacin, and tigecycline (Figure 6B). Collectively, these findings indicate that all *Providencia* isolates recovered from dead crocodiles in Hainan Province displayed multidrug‐resistant phenotypes, although the resistance patterns varied considerably among species.

### 3.5. Pathogenicity Analysis

#### 3.5.1. Distribution of VGs

Next, we investigated the prevalence of VGs among the 65 *Providencia* isolates, targeting nine genes involved in four major pathogenic processes: adhesion and outer membrane‐associated functions (*papC*, *papD*, *fimC*, and *ompA*), iron acquisition (*ireA*, *fepC*, and *fptA*), motility (*motA*), and efflux‐associated function (*acrA*). Overall, the detection rates varied considerably across the gene panel. The *ompA* gene was universally present in all isolates (100%, 65/65), representing the most prevalent virulence‐associated determinant. The *fepC* and *motA* genes were detected at moderate frequencies of 57% (37/65) and 51% (33/65), respectively, while *acrA* was also identified in 51% (33/65) of the isolates. In contrast, the adhesion‐related genes were rarely detected: *papC* was found in only 9% (6/65) of isolates, whereas *papD* and *fimC* were each detected in only 3% (2/65). Among the iron acquisition genes, *ireA* and *fptA* showed detection rates of 20% (13/65) and 3% (2/65), respectively (Figure 5).

Marked species‐associated differences were evident in the distribution of VGs. Among the 18 *P. stuartii* isolates from Dongfang, *ompA* was universally present (100%, 18/18), and *acrA* was detected at a high frequency of 94% (17/18), followed by *fepC* and *motA*, each at 39% (7/18). Among the 14 *P. hangzhouensis* isolates (two from Baisha Li Autonomous County and 12 from Dongfang), *ompA* was also detected in all isolates (100%, 14/14), whereas *fepC* and *motA* were each detected in 57% (8/14) of the strains. The 31 *P. rettgeri* isolates from Dongfang exhibited a broader virulence gene profile, with universal carriage of *ompA* (100%, 31/31), followed by *fepC* (65%, 20/31), *motA* (52%, 16/31), *ireA* (35%, 11/31), and *acrA* (35%, 11/31). The two *P. alcalifaciens* isolates from Chengmai displayed the most extensive virulence gene repertoire, carrying all nine tested genes except *ireA*, with 100% positivity for the remaining eight genes. Notably, the two *P. alcalifaciens* isolates from Chengmai and the two *P. hangzhouensis* isolates from Baisha Li Autonomous County harbored more VGs (eight and six genes, respectively) than the isolates from Dongfang (1–5 genes) (Figure 5). Together, these findings indicate that virulence gene carriage among *Providencia* isolates from deceased crocodiles in Hainan was widespread but highly variable across species and geographic origins, with certain strains harboring a more extensive virulence repertoire.

#### 3.5.2. Zebrafish Challenge Assay

**Figure 7:**
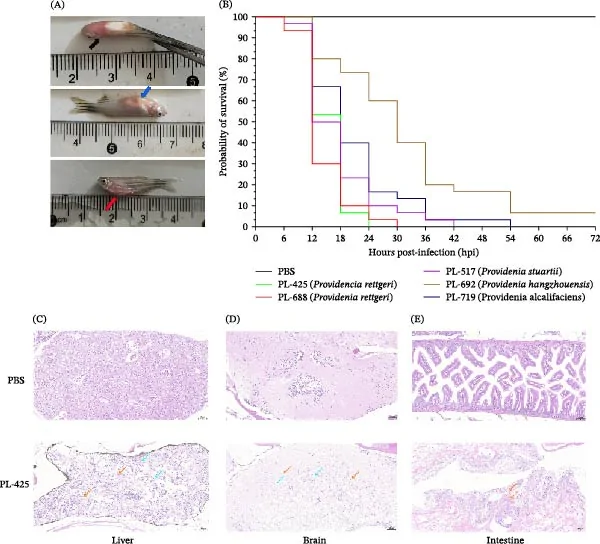
Pathogenicity of representative *Providencia* isolates in zebrafish and histopathological lesions induced by *P. rettgeri* PL‐425. (A) Representative clinical signs in zebrafish after intraperitoneal challenge with *Providencia* isolates, including gill hemorrhage (black arrow), severe abdominal distension (blue arrow), and marked abdominal congestion (red arrow). (B) Survival curves of zebrafish challenged with five representative *Providencia* strains, including PL‐425 (*Providencia rettgeri*), PL‐688 (*Providencia rettgeri*), PL‐517 (*Providencia stuartii*), PL‐692 (*Providencia hangzhouensis*), and PL‐719 (*Providencia alcalifaciens*). Zebrafish injected with sterile PBS served as the control group. (C–E) Histopathological lesions in the liver (C), brain (D), and intestine (E) of zebrafish challenged with the highly virulent strain *P. rettgeri* PL‐425. The upper panels show tissues from PBS‐injected control fish, and the lower panels show tissues from PL‐425–challenged fish. In the liver, hepatocellular swelling and cytoplasmic vacuolation are indicated by cyan arrows, and hepatocellular necrosis with pyknotic or karyorrhectic nuclei is indicated by orange arrows. In the brain, neuronal edema and cytoplasmic vacuolation are indicated by cyan arrows, and necrotic neurons are indicated by orange arrows. In the intestine, mild necrosis of villous epithelial cells is indicated by orange arrows.

Based on their biological characteristics, isolation sources, and virulence gene profiles, five representative *Providencia* strains were selected for pathogenicity assessment in zebrafish by intraperitoneal injection, including two *P. rettgeri* strains and one strain each of *P. stuartii*, *P. alcalifaciens*, and *P. hangzhouensis*. The main clinical signs observed in infected zebrafish included gill hemorrhage (Figure 7A, black arrows), severe abdominal distension (Figure 7A, blue arrows), and marked abdominal congestion (Figure 7A, red arrows). Most zebrafish in the challenged groups died within 36 h postinfection.

Notably, the two *P. rettgeri* strains (PL‐425 and PL‐688) exhibited the highest virulence, causing 100% mortality within 24 h and 30 h, respectively. The *P. stuartii*–challenged group reached 100% mortality within 42 h, whereas the *P. alcalifaciens*–challenged group reached 100% mortality at 54 h postinfection. In contrast, the *P. hangzhouensis* strain showed the lowest virulence, with two zebrafish surviving at 72 h postinfection, corresponding to a survival rate of 6.7% (2/30) (Figure 7B). These findings indicate that the *Providencia* strains isolated from deceased crocodiles generally exhibited high pathogenicity in zebrafish, with clear differences in virulence among the four *Providencia* species. Among them, *P. rettgeri* showed relatively stronger pathogenic potential.

#### 3.5.3. Histopathological Analysis

Histopathological examination was performed on the liver, intestine, and brain of zebrafish that died after challenge with the most virulent *Providencia* strain, *P. rettgeri* PL‐425. In the liver, the normal hepatic architecture was disrupted, with irregular arrangement of hepatic cords. Extensive hepatocellular swelling and cytoplasmic vacuolation were observed (Figure 7C, cyan arrows), together with numerous hepatocytes showing necrotic changes characterized by pyknotic and karyorrhectic nuclei (Figure 7C, orange arrows).

In the brain, widespread neuronal edema was observed, characterized by cellular swelling and cytoplasmic vacuolation (Figure 7D, cyan arrows), along with a small number of neurons showing necrotic changes, including pyknosis and karyorrhexis (Figure 7D, orange arrows). In the intestine, the villi remained elongated and finger‐like, and the villous epithelium consisted mainly of simple columnar epithelial cells and abundant goblet cells. A small number of villous epithelial cells exhibited necrotic changes with pyknotic and karyorrhectic nuclei (Figure 7E, orange arrows). The lamina propria was composed of connective tissue, and the muscular layer remained well‐defined with regularly arranged myocytes. Notably, goblet cells appeared increased, accompanied by mild necrosis of villous epithelial cells.

These histopathological findings indicate that infection with the crocodile‐derived highly virulent *P. rettgeri* strain PL‐425 caused marked multiorgan injury in zebrafish, including hepatocellular necrosis and vacuolation, neuronal edema and necrosis, and intestinal epithelial cell necrosis with goblet cell hyperplasia. These pathological changes partially mirrored the severe multiorgan lesions observed in dead farmed crocodiles, further supporting the high pathogenic potential of this strain.

### 3.6. Whole‐Genome Analysis of the Highly Virulent *P. rettgeri* Strain PL‐425

To further elucidate the pathogenic basis of the highly virulent *P. rettgeri* strain PL‐425, whole‐genome sequencing was performed. The genome was 4,598,886 bp in length, with a GC content of 40.23%. A total of 4282 open reading frames (ORFs) were predicted, with an average length of 923 bp, accounting for 85.96% of the genome. ncRNA analysis identified 71 tRNAs (5540 bp), as well as 16S rRNA (1063 bp) and 23S rRNA (1856 bp) (Figure 8A). The genome sequence data for strain PL‐425 have been deposited in the NCBI database under BioSample Accession Number SAMN52019661.

Figure 8Whole‐genome characteristics and functional annotation of the highly virulent *Providencia rettgeri* strain PL‐425. (A) Circular genome map of strain PL‐425. Rings from the outside to the center represent genome size, protein‐coding genes on the forward strand, protein‐coding genes on the reverse strand, tRNA and rRNA genes, genomic islands, GC content, and GC skew. Protein‐coding genes are colored according to their COG functional categories. (B) Histogram showing the COG functional classification of predicted coding sequences in the PL‐425 genome. (C) GO annotation of predicted genes in the PL‐425 genome, grouped into biological process, cellular component, and molecular function categories. (D) Pie charts showing the distribution of virulence‐associated genes predicted by VFDB, including genes related to invasion, immune modulation, biofilm, adherence, antimicrobial activity/competitive advantage, regulation, motility, effector delivery systems, exoenzymes, stress survival, posttranslational modification, nutritional/metabolic factors, and exotoxins.

**Figure:**
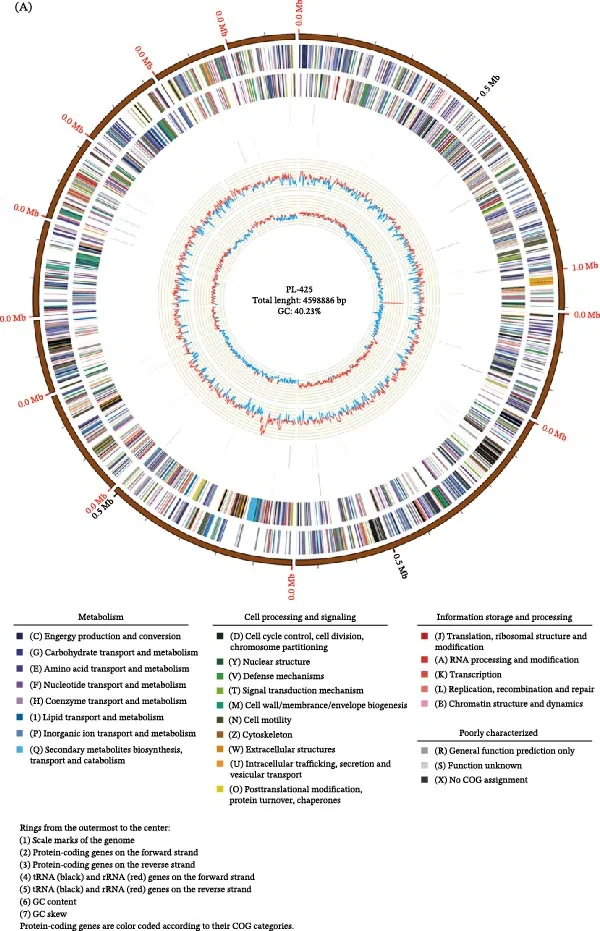

**Figure:**
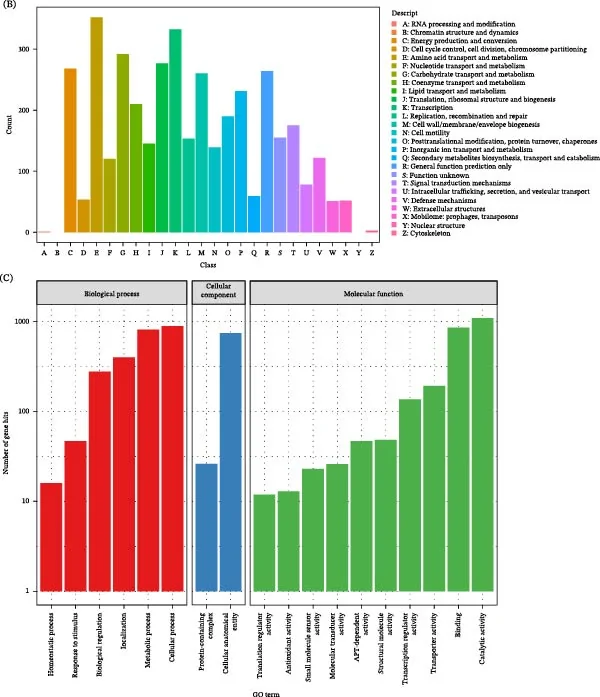

**Figure:**
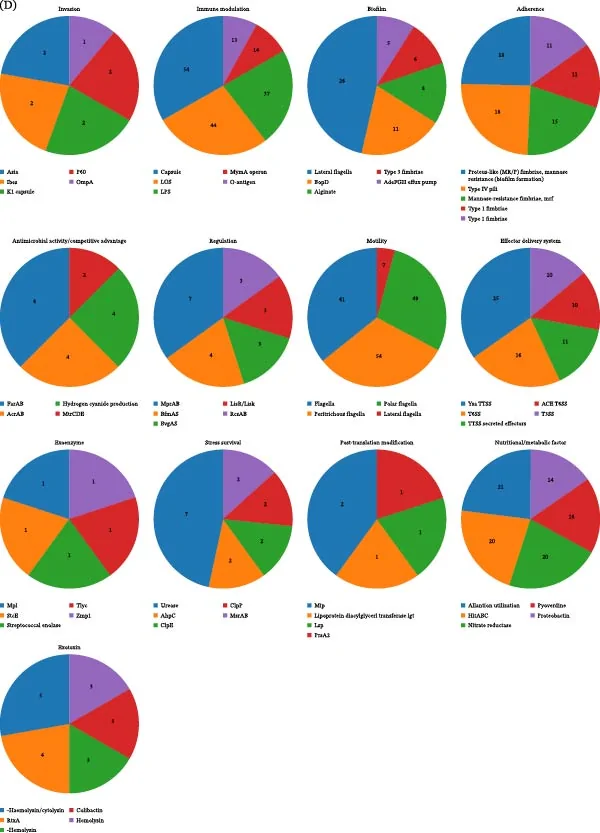

COG and GO functional annotation showed that the coding sequences were predominantly associated with genetic information processing, including translation, transcription, replication, and repair, as well as core metabolic processes such as amino acid, carbohydrate, and energy metabolism, supporting the environmental adaptability and pathogenic potential of this strain (Figure 8B,C).

VFDB analysis identified multiple categories of VGs. Motility‐related genes, particularly those associated with flagella (61 genes), and immune modulation–related genes, particularly capsule‐associated genes (54 genes), were the most abundant and may contribute substantially to pathogenicity. Additional virulence‐related determinants included adherence‐associated genes, such as MR/P fimbriae and type IV pili (18 genes each) and toxin‐related genes, including α‐haemolysin/cytolysin (five genes) and *rtxA* (four genes) (Figure 8D). Together, these genomic features provide a plausible genetic basis for motility, immune evasion, host adhesion, and host cell damage, consistent with the high virulence of this strain.

## 4. Discussion

This study provides a systematic view of *Providencia* infection associated with mortality in farmed Siamese crocodiles in Hainan Province. Earlier reports in crocodilians mainly consisted of individual or small‐scale case descriptions of *P. rettgeri* meningitis or septicemia in hatchling saltwater crocodiles, juvenile American alligators, and farmed crocodiles [3–5]. In contrast, the present work combined pathology, species‐level identification, phenotypic characterization, antimicrobial resistance profiling, zebrafish challenge, and whole‐genome analysis, showing that crocodile‐associated *Providencia* populations are not only taxonomically diverse but also phenotypically heterogeneous and frequently multidrug‐resistant. Given the expanding and taxonomically dynamic nature of the genus *Providencia*, these findings are important for both disease surveillance and risk assessment in crocodile farming systems [7, 12].

The necropsy and histopathological findings strongly support the interpretation that the affected crocodiles died from a systemic septicemic process rather than from localized opportunistic colonization. Although *P. rettgeri*–associated meningitis or meningoencephalitis has been reported previously in hatchling saltwater crocodiles and juvenile farmed American alligators [3, 4], no definitive histopathological evidence of meningitis was observed in the examined Siamese crocodiles in the present study. Severe serous effusions, gastrointestinal congestion, hepatic degeneration, renal tubular injury, splenic necrosis, and intestinal epithelial damage are all compatible with disseminated bacterial infection, and the recovery of *Providencia* from multiple internal organs and heart blood further strengthens this interpretation. Notably, *P. rettgeri* was the predominant species in the present collection, which is consistent with previous crocodilian reports in which *P. rettgeri* was the principal pathogenic species recovered from diseased animals [3–5]. At the same time, the recovery of *P. stuartii*, *P. hangzhouensis*, and *P. alcalifaciens* expands the known diversity of crocodile‐associated *Providencia* and suggests that multiple members of the genus may participate in crocodile farm disease complexes. More broadly, intensive crocodilian farming is known to favor stress‐associated infectious syndromes, including septicemic conditions, which provides an important ecological background for the emergence of such opportunistic pathogens in farm settings [2].

A notable phenotypic feature of the isolate collection was the predominance of strong motility but mostly weak or moderate biofilm formation. This pattern suggests that, in the context of acute crocodile infection, rapid migration, tissue dissemination, and host interaction may be more important than robust static biofilm formation on abiotic surfaces. This interpretation is biologically plausible because *Providencia* and related Proteeae are characteristically motile organisms, and work on *P. stuartii* has shown that swarming capacity is accompanied by measurable adhesion and invasion of epithelial cells [44]. In addition, comparative genomic analysis across the genus has demonstrated substantial diversity in loci related to flagella, fimbriae, resistance, and environmental adaptation, indicating that the balance between motility, surface attachment, and persistence may differ considerably among species and strains [45]. Therefore, the relatively weak biofilm phenotype of many crocodile‐derived isolates should not be interpreted as evidence of low pathogenicity; rather, it may reflect a virulence strategy biased toward motility‐driven colonization and rapid extraintestinal spread.

The antimicrobial resistance results are among the most important practical findings of this study. All isolates fulfilled the definition of MDR, and resistance determinants were widespread, with universal carriage of *arnA*, *tetA*, and *cpxA*; very high detection rates of *rpsL* and *qnrD*; and frequent carriage of class 1 integrons. According to information provided by the farm owners and available farm management records, tetracycline‐class antibiotics, mainly oxytetracycline, doxycycline, and tetracycline, had been routinely used in these farms for disease prevention or treatment. Consistent with this practice, most crocodile‐derived *Providencia* isolates showed resistance to tetracycline‐class antibiotics, with resistance rates of 91% for doxycycline, tetracycline, and oxytetracycline. In addition, *tetA* was detected in all 65 isolates. These findings suggest that routine or repeated exposure to tetracycline‐class antibiotics may have contributed to the selection and maintenance of tetracycline‐resistant *Providencia* strains in these farms. However, because detailed quantitative records of antibiotic dosage, frequency, duration, and treatment history were not available, this association should be interpreted cautiously and cannot be considered direct evidence of causality. This profile is consistent with previous work showing that *Providencia* species often accumulate diverse resistance genes and mobile elements and that integrons are important contributors to resistance dissemination in Proteeae [22, 45, 46]. Phenotypically, the universal resistance to polymyxin B is not unexpected because *Providencia* is widely regarded as intrinsically resistant to polymyxins, and polymyxin susceptibility testing itself requires methodological caution [47]. The tigecycline results should likewise be interpreted carefully: although many isolates in the present study appeared susceptible, tigecycline MIC testing is known to be influenced by medium freshness, and *Providencia* has reduced in vitro susceptibility to tigecycline compared with many other Enterobacterales [21, 48]. Taken together, these results indicate that crocodile farms may constitute reservoirs of multidrug‐resistant *Providencia*, with clear implications for treatment choice and antimicrobial stewardship.

From a conservation perspective, the detection of highly virulent and multidrug‐resistant *P. rettgeri* in farmed *C. siamensis* raises concerns about the role of captive breeding facilities as potential reservoirs of AMR pathogens. The high prevalence of class 1 integrons suggests that mobile genetic elements may facilitate the horizontal transfer of resistance determinants within and beyond farm environments. These findings underscore the importance of integrating systematic pathogen surveillance and antimicrobial stewardship into the conservation management framework for farmed *C. siamensis*, consistent with a One Health approach.

The virulence‐gene survey and zebrafish challenge experiments further support the pathogenic significance of the crocodile‐derived isolates. The universal presence of *ompA* and the relatively frequent detection of *fepC*, *motA*, and *acrA* suggest that outer membrane integrity, iron acquisition, motility, and efflux‐associated stress adaptation are more conserved in these isolates than classical fimbrial genes such as *papC*, *papD*, and *fimC*. This is biologically reasonable because successful systemic infection depends not only on adhesion but also on survival under iron limitation, resistance to host defenses, and effective dissemination in tissues. Previous studies have shown that *P. stuartii* can adhere to and invade epithelial cells, whereas *P. alcalifaciens* virulence can depend strongly on lipopolysaccharide‐associated resistance to host antimicrobial peptides and oxidative stress [44, 49]. In the present study, all five representative isolates showed clear pathogenicity in zebrafish, and the two *P. rettgeri* strains were the most virulent, which agrees with the dominant role of *P. rettgeri* in previous crocodilian disease reports [3–5]. Moreover, the multiorgan lesions induced by PL‐425 in zebrafish partially mirrored those observed in the dead crocodiles, strengthening the inference that these isolates were true pathogens rather than incidental colonizers. Similar pathogenic behavior has also been documented for *P. rettgeri* in turtles and shrimp, supporting the broader host versatility and virulence potential of this species [50, 51].

The whole‐genome analysis of *P. rettgeri* PL‐425 provides a plausible genetic explanation for its high virulence. Its genome size and GC content are broadly comparable to those reported for other *Providencia* genomes, while the enrichment of flagellar genes, capsule‐associated genes, MR/P fimbriae, type IV pili, α‐haemolysin/cytolysin, and *rtxA* suggests a multifactorial virulence architecture involving motility, host adhesion, immune evasion, and tissue injury. Comparative genomic analyses have shown that the genus *Providencia* has an open pan‐genome and substantial variation in virulence‐ and resistance‐associated accessory genes, which may explain why some crocodile‐derived strains in this study were much more pathogenic than others [12, 45]. Likewise, genomic work on turtle‐derived pathogenic *P. rettgeri* identified multiple adhesion‐, toxin‐, hemolysin‐, and resistance‐associated loci, consistent with the genomic profile observed here for PL‐425 [50]. Thus, the high virulence of PL‐425 is likely to result from the combined action of multiple colonization, persistence, and damage‐associated systems rather than from any single determinant.

From an epidemiological and economic perspective, the detection of highly virulent and multidrug‐resistant *P. rettgeri* in farmed *C. siamensis* confirms its status as an emerging pathogen of concern in reptile farming. The high prevalence of class 1 integrons suggests that mobile genetic elements may facilitate the spread of resistance, amplifying the biosecurity risks within and potentially beyond farm environments. These findings align with recent reports of *P. rettgeri* causing bacteremia in farmed rat snakes [6], indicating a broader emerging pattern. This study underscores the critical importance of integrating systematic pathogen surveillance and robust antimicrobial stewardship into the management of crocodile farming to mitigate the economic impact of this emerging disease.

Despite these findings, several limitations should be acknowledged. The number of crocodiles and farms included in this study was still limited, and some species‐level groups contained only a small number of isolates. In addition, whole‐genome sequencing was performed for only one highly virulent strain, and the VGs identified in this study were not functionally validated. Given the substantial genomic diversity, resistance heterogeneity, and taxonomic complexity reported within the genus *Providencia*, broader epidemiological surveys, comparative genomic analyses of additional representative isolates, and targeted functional studies will be needed to further clarify the transmission, virulence mechanisms, and resistance evolution of crocodile‐derived *Providencia* [12, 45]. Nevertheless, the present study provides a useful basis for future genome‐based surveillance and improved disease management in crocodile farming systems.

## 5. Conclusion

In conclusion, crocodile‐derived *Providencia* isolates were diverse, multidrug‐resistant, and highly pathogenic, with *P. rettgeri* representing the dominant and relatively more virulent species. These findings improve the current understanding of *Providencia* in Siamese crocodiles and provide a basis for future surveillance and disease control in crocodile farming. Given the conservation status of *C. siamensis*, integrated pathogen surveillance and antimicrobial stewardship should be prioritized in captive breeding programs to safeguard both farmed populations and the species’ long‐term conservation value.

## Author Contributions

Mengqi Wang performed the investigation and wrote the original draft. Caike Li conducted the formal analysis. Yixin Tian provided resources and contributed to visualization. Shuai Ye and Jiahao Yu were responsible for validation. Zhongtao Liu contributed to funding acquisition and resources. Guiying Guo managed the software. Lixia Fan and Nuo Yang contributed to visualization. Jifeng Zeng and Jiping Zheng participated in funding acquisition and managed the software. Xuesong Li conducted the formal analysis and contributed to writing – original draft.

## Funding

This study was sponsored by the Key Research and Development Program of Hainan Province (Grants ZDYF2024XDNY198, ZDYF2024SHFZ142, and ZDYF2026SHFZ045), the National Natural Science Foundation of China (Grant 32360879), and the Innovative Research Projects for Postgraduates in Hainan Province (Grant Hyb2025‐137).

## Disclosure

All AI‐assisted suggestions were critically reviewed and revised by the authors to preserve the original intended meaning. All authors read, reviewed, and approved the final manuscript.

## Ethics Statement

This study was conducted as per the recommendations for the care and use of laboratory animals. The animal experimental protocol was carried out according to the Hainan University Application for Animal Welfare and Ethical Review (HNUAUCC‐2023‐00166). All animal experiments were performed in strict accordance with the ARRIVE guidelines 2.0. A completed ARRIVE 2.0 Full Checklist is available as Supporting Information 2: File S1. There are no human participants in this study.

## Conflicts of Interest

The authors declare no conflicts of interest.

## Supporting Information

Additional supporting information can be found online in the Supporting Information section.

## Supporting information


**Supporting Information 1** Table S1: The primers used for amplification of 16S rDNA and *fusA*, detection of antimicrobial resistance genes and class 1 integrons, and screening of virulence‐associated genes in crocodile‐derived *Providencia* isolates.

**Supporting Information 2** File S1: The completed ARRIVE 2.0 Full Checklist for the animal experiments conducted in this study.

## Data Availability

All raw datasets generated and analyzed during this study, including original quantitative values for all figures and tables, drug susceptibility test results, phenotypic characterization data, molecular experiment records, full‐size published images, and histopathological analysis data, are publicly available in the Figshare repository under a CC‐BY 4.0 open license [52]. The permanent DOI is https://doi.org/10.6084/m9.figshare.32061495. The genome sequence data for strain PL‐425 have been deposited in the NCBI database under BioSample Accession Number SAMN52019661.
