# Supplementary material for: Multidrug‐Resistant Providencia rettgeri: An Emerging Pathogen in Farmed Siamese Crocodiles (Crocodylus siamensis) in Hainan, China
Source: Transbound Emerg Dis. 2026 Aug 26;2026:5761973. doi: 10.1155/tbed/5761973 (PMC13519305; doi:10.1155/tbed/5761973)
Supplement: Supplementary file 1 [file TBED-2026-5761973-s002.pdf]

NOTE: Please save this file locally before filling in the table, DO NOT work on the file within your internet browser as changes will not be saved. Adobe Acrobat Reader (available free here) is recommended for completion.

ARRIVE

The ARRIVE guidelines 2.0: author checklist

The ARRIVE Essential 10

These items are the basic minimum to include in a manuscript. Without this information, readers and reviewers cannot assess the reliability of the findings.

Item		Recommendation	Section/line number, or reason for not reporting
**Study design**	1	For each experiment, provide brief details of study design including:	
		a. The groups being compared, including control groups. If no control group has been used, the rationale should be stated.	Section 2.4.2; 5 bacterial challenge groups (PL-425, PL-688, PL-517, PL-692, PL-719) + 1 sterile PBS negative control group
		b. The experimental unit (e.g. a single animal, litter, or cage of animals).	Section 2.4.2; Single individual zebrafish
**Sample size**	2	a. Specify the exact number of experimental units allocated to each group, and the total number in each experiment. Also indicate the total number of animals used.	Section 2.4.2; 30 zebrafish per experimental group, total 180 zebrafish used in the virulence challenge assay
		b. Explain how the sample size was decided. Provide details of any *a priori* sample size calculation, if done.	No a priori sample size calculation was performed; sample size was determined based on standard practices widely adopted in similar aquatic animal bacterial virulence studies
**Inclusion and exclusion criteria**	3	a. Describe any criteria used for including and excluding animals (or experimental units) during the experiment, and data points during the analysis. Specify if these criteria were established *a priori*. If no criteria were set, state this explicitly.	Section 2.4.2; Inclusion criteria (established a priori): Clinically healthy adult zebrafish, body weight 0.35 ± 0.05 g, acclimatized for ≥7 days with pre-experimental mortality ≤2%; No exclusion criteria were established
		b. For each experimental group, report any animals, experimental units or data points not included in the analysis and explain why. If there were no exclusions, state so.	No animals, experimental units or data points were excluded from any analysis
		c. For each analysis, report the exact value of *n* in each experimental group.	Section 2.4.2; n=30 per group for 72-hour survival analysis; n=3 per group for histopathological analysis
**Randomisation**	4	a. State whether randomisation was used to allocate experimental units to control and treatment groups. If done, provide the method used to generate the randomisation sequence.	Simple randomisation was used to allocate zebrafish to groups; random number sequences were generated using a computer program
		b. Describe the strategy used to minimise potential confounders such as the order of treatments and measurements, or animal/cage location. If confounders were not controlled, state this explicitly.	All zebrafish were housed under identical environmental conditions (temperature, water quality, light cycle); the order of bacterial injection was randomised to minimise temporal confounding
**Blinding**	5	Describe who was aware of the group allocation at the different stages of the experiment (during the allocation, the conduct of the experiment, the outcome assessment, and the data analysis).	Blinding was not implemented at any stage of the experiment (allocation, conduct, outcome assessment, data analysis) due to practical operational constraints in the laboratory setting
**Outcome measures**	6	a. Clearly define all outcome measures assessed (e.g. cell death, molecular markers, or behavioural changes).	Section 2.4.2; Outcome measures: 72-hour cumulative mortality rate, clinical signs (gill hemorrhage, abdominal distension, abdominal congestion), histopathological lesions in liver, brain and intestine
		b. For hypothesis-testing studies, specify the primary outcome measure, i.e. the outcome measure that was used to determine the sample size.	Primary outcome measure: 72-hour cumulative mortality rate post-infection
**Statistical methods**	7	a. Provide details of the statistical methods used for each analysis, including software used.	Section 3.5.2; Survival curves were generated using the Kaplan-Meier method; All statistical analyses were performed using GraphPad Prism 9.0 software
		b. Describe any methods used to assess whether the data met the assumptions of the statistical approach, and what was done if the assumptions were not met.	Normality testing was not performed; non-parametric Kaplan-Meier survival analysis was used, which is appropriate for time-to-event data and does not require normality assumptions
**Experimental animals**	8	a. Provide species-appropriate details of the animals used, including species, strain and substrain, sex, age or developmental stage, and, if relevant, weight.	Section 2.4.2; Species: Danio rerio (zebrafish); Strain: Wild-type; Developmental stage: Adult; Body weight: 0.35 ± 0.05 g; Sex was not determined for individual animals
		b. Provide further relevant information on the provenance of animals, health/immune status, genetic modification status, genotype, and any previous procedures.	Section 2.4.2; Provenance: Local commercial aquaculture farm in Haikou, Hainan, China; Health status: Clinically healthy with no signs of disease; Genetic modification status: Non-genetically
**Experimental procedures**	9	For each experimental group, including controls, describe the procedures in enough detail to allow others to replicate them, including:	
		a. What was done, how it was done and what was used.	Section 2.4.2; Zebrafish were briefly anesthetized by immersion in ice water for approximately 3 seconds, then intraperitoneally injected with 10 μL of
		b. When and how often.	Section 2.4.2; Mortality and clinical signs were recorded every 6 hours for a total of 72 hours post-infection
		c. Where (including detail of any acclimatisation periods).	Section 2.4.2; Experiments were conducted at the Laboratory of Infection and Immunology, Hainan University; Acclimatisation period: ≥7 days in
		d. Why (provide rationale for procedures).	Section 1 and 2.4.2; Zebrafish is a well-established and widely accepted model for assessing the virulence of aquatic bacterial pathogens; Intraperitoneal injection
**Results**	10	For each experiment conducted, including independent replications, report:	
		a. Summary/descriptive statistics for each experimental group, with a measure of variability where applicable (e.g. mean and SD, or median and range).	Section 3.5.2; Cumulative mortality rates are presented as percentages; Survival curves show the proportion of surviving animals over the 72-hour observation period
		b. If applicable, the effect size with a confidence interval.	Effect sizes and 95% confidence intervals were not calculated for this study

The Recommended Set

These items complement the Essential 10 and add important context to the study. Reporting the items in both sets represents best practice.

Item		Recommendation	Section/line number, or reason for not reporting
**Abstract**	11	Provide an accurate summary of the research objectives, animal species, strain and sex, key methods, principal findings, and study conclusions.	Abstract section; Includes all required elements
**Background**	12	a. Include sufficient scientific background to understand the rationale and context for the study, and explain the experimental approach.<br>b. Explain how the animal species and model used address the scientific objectives and, where appropriate, the relevance to human biology.	Section 1 (Introduction); Provides comprehensive background on Providencia infections in crocodilians, antimicrobial resistance concerns, and the rationale for the experimental approach used<br>Section 1; The zebrafish model allows rapid, high-throughput assessment of bacterial virulence, which addresses the objective of comparing pathogenicity across different Providencia isolates; Findings are relevant to
**Objectives**	13	Clearly describe the research question, research objectives and, where appropriate, specific hypotheses being tested.	Section 1 (last paragraph); Clearly states the re
**Ethical statement**	14	Provide the name of the ethical review committee or equivalent that has approved the use of animals in this study, and any relevant licence or protocol numbers (if applicable). If ethical approval was not sought or granted, provide a justification.	Declarations > Ethics approval and consent to participate; Approved by the Animal Welfare and Ethics Committee of Hainan University (Approval No. HNUAUCC-2023-000013)
**Housing and husbandry**	15	Provide details of housing and husbandry conditions, including any environmental enrichment.	Section 2.4.2; Housing conditions: Dechlorinate
**Animal care and monitoring**	16	a. Describe any interventions or steps taken in the experimental protocols to reduce pain, suffering and distress.<br>b. Report any expected or unexpected adverse events.<br>c. Describe the humane endpoints established for the study, the signs that were monitored and the frequency of monitoring. If the study did not have humane endpoints, state this.	Section 2.4.2; Ice water anesthesia was used to minimize pain and distress during the intraperitoneal injection procedure<br>No expected or unexpected adverse events occurred during the experiment<br>Formal pre-defined humane endpoints were not established for this study; All surviving and moribund animals were euthanized by immersion in ice water at the end of the 72-hour observation period
**Interpretation/ scientific implications**	17	a. Interpret the results, taking into account the study objectives and hypotheses, current theory and other relevant studies in the literature.<br>b. Comment on the study limitations including potential sources of bias, limitations of the animal model, and imprecision associated with the results.	Section 4 (Discussion); Interprets all key findings in the context of existing literature on Providencia virulence, antimicrobial resistance, and crocodile farming diseases<br>Section 4 (last paragraph); Explicitly discusses study limitations: limited number of crocodiles and farms, small sample size for some species groups, whole-genome sequencing performed on only one strain,
**Generalisability/ translation**	18	Comment on whether, and how, the findings of this study are likely to generalise to other species or experimental conditions, including any relevance to human biology (where appropriate).	Section 4; Discusses that findings are generalisable to other crocodile farming regions and relevant to public health, as multidrug-resistant Providencia species can cause opportunistic infections in humans
**Protocol registration**	19	Provide a statement indicating whether a protocol (including the research question, key design features, and analysis plan) was prepared before the study, and if and where this protocol was registered.	The study protocol was not registered in any public database
**Data access**	20	Provide a statement describing if and where study data are available.	Declarations > Availability of data and materials
**Declaration of interests**	21	a. Declare any potential conflicts of interest, including financial and non-financial. If none exist, this should be stated.<br>b. List all funding sources (including grant identifier) and the role of the funder(s) in the design, analysis and reporting of the study.	Declarations > Competing interests; The authors declare that they have no competing interests<br>Declarations > Funding; Lists all funding sources with corresponding grant numbers; Explicitly states that the funders had no role in study design, data collection and analysis, decision to publish, or preparation of the
