# Supplementary material for: Multidrug‐Resistant Providencia rettgeri: An Emerging Pathogen in Farmed Siamese Crocodiles (Crocodylus siamensis) in Hainan, China
Source: Transbound Emerg Dis. 2026 Aug 26;2026:5761973. doi: 10.1155/tbed/5761973 (PMC13519305; doi:10.1155/tbed/5761973)
Supplement: Supplementary file 2 [file TBED-2026-5761973-s001.docx]

**Table S1**. **PCR primes used in the study**

1) Primers used for amplification of 16S rDNA and *fusA*.

| Gene | Primer sequence  (5′-3′) | Product size (bp) | Tm  (℃) |
| --- | --- | --- | --- |
| 16S rDNA | AGAGTTTGATCAGGCTCAG CGGTTACCTTGTTACGACTT | 1465 | 55.84  54.90 |
| *fusA* | TTGCAACAGAGATTACTGGCTC  TCTATACTGGTGTAAACCATA | 1164 | 58.66  50.70 |

2) Primers used for detection of antibiotic resistance genes.

| Gene | Primer sequence  (5′-3′) | Product size  (bp) | Tm  (℃) |
| --- | --- | --- | --- |
| *tetA* | CATTGTCGGCTTTGTCGTGG  TCATCGCACCAATCAGCCAT | 290 | 57.84  57.90 |
| *mexI* | GTTCCCCCAAAACTAGCGGA  CGCACTGATGATGGCTTTCG | 708 | 56.31  59.55 |
| *cat* | GGCTACTATCACGGCCACTC  CGACTACCTATCACGGCACC | 323 | 57.70  57.65 |
| *floR* | GCCTTTGTTGCGTTTCGTCT  CGCGAAGGCCAAGCTAAATC | 456 | 57.52  59.56 |
| *rpsL* | ACGTGGCGTATGTACTCGTG  TCGCGCCGTATTTAGAACGA | 269 | 57.83  59.09 |
| *cpxA* | ATGACCTACTTTGGTGGCGG  CTCTGGGTGTGGCCGTAAAT | 443 | 57.31  57.26 |
| *bla_TEM_* | TTCCGTGTCGCCCTTATTCC  CAATGATACCGCGAGACCCA | 718 | 57.03  57.82 |
| *ampC* | GTGCCGGAGACCATTACGAA  CTTCAGGGATAAAGGCGGCA | 634 | 57.69  58.52 |
| *arnA* | TTACTCCTCAGTTGCGCGTT  AACTTCCGCTGCTTCACGTA | 391 | 58.31  58.31 |
| *qnrD* | TCACGAGATCAATTTACGGGG  GCCATTCCAGCGATTTTCCC | 399 | 55.96  57.00 |
| *intI1* | AACTGCGGGTCAAGGATCTG | 360 | 60.04 |
|  | TCGTGATGCCTGCTTGTTCT |  | 59.96 |

3) Primers used for detection of virulence genes

| Gene | Primer sequence  (5′-3′) | Product size (bp) | Tm  (℃) |
| --- | --- | --- | --- |
| *papC* | GTGGGTACGACCGTTGTTGA  GTGGCTCAGCACTTGACTCT | 421 | 55.64  58.14 |
| *ireA* | TCAGTGCATCAGGCTTCTCG  ATAATAGCCCCAGTGGTGACG | 421 | 59.7  57.05 |
| *fptA* | ACTGCTCCATGGGCTTTATC  CATAGGTACGCCAGCCATATT | 744 | 55.63  56.44 |
| *fimC* | CATTAGGTGCAACGCGAGTG  CGGCAGTTGGTTATTGGTCG | 221 | 58.79  57.19 |
| *papD* | GAATCCTGCATTTAGTGGGATCA  ACTCGGCATGACGAAAAATCA | 344 | 56.59  56.72 |
| *motA* | TTTCGAGCGCAAGAAGACCT  TGGCTATGTCATGGTCGGTG | 248 | 59.17  57.03 |
| *fepC* | GGGCTTACACCGCATTTGTC  ATGCTGCTTCAACCCTTGGA | 264 | 56.38  57.27 |
| *ompA* | TATCGCAGTAGCAGTGGCAG  CCGTTGTCTGGACGAGTACC | 573 | 59.73  56.96 |
| *acrA* | TAGACGCTTATCTCGTGCCG  CGTGGCTTCTCCGTGATAGT | 535 | 59.78  58.08 |
